# *Vibrio cholerae* Infection of *Drosophila* *melanogaster* Mimics the Human Disease Cholera

**DOI:** 10.1371/journal.ppat.0010008

**Published:** 2005-09-30

**Authors:** Nathan S Blow, Robert N Salomon, Kerry Garrity, Isabelle Reveillaud, Alan Kopin, F. Rob Jackson, Paula I Watnick

**Affiliations:** 1 Department of Geographic Medicine and Infectious Diseases, Tufts-New England Medical Center, Boston, Massachusetts, United States of America; 2 Department of Pathology, Tufts-New England Medical Center, Boston, Massachusetts, United States of America; 3 Molecular Cardiology Research Institute, Tufts-New England Medical Center, Boston, Massachusetts, United States of America; 4 Department of Neurosciences, Tufts University School of Medicine, Boston, Massachusetts, United States of America; Stanford University, United States of America

## Abstract

Cholera, the pandemic diarrheal disease caused by the gram-negative bacterium *Vibrio cholerae,* continues to be a major public health challenge in the developing world*.* Cholera toxin, which is responsible for the voluminous stools of cholera, causes constitutive activation of adenylyl cyclase, resulting in the export of ions into the intestinal lumen. Environmental studies have demonstrated a close association between *V. cholerae* and many species of arthropods including insects. Here we report the susceptibility of the fruit fly, *Drosophila melanogaster,* to oral *V. cholerae* infection through a process that exhibits many of the hallmarks of human disease: (i) death of the fly is dependent on the presence of cholera toxin and is preceded by rapid weight loss; (ii) flies harboring mutant alleles of either adenylyl cyclase, Gsα, or the Gardos K^+^ channel homolog SK are resistant to *V. cholerae* infection; and (iii) ingestion of a K^+^ channel blocker along with *V. cholerae* protects wild-type flies against death. In mammals, ingestion of as little as 25 μg of cholera toxin results in massive diarrhea. In contrast, we found that ingestion of cholera toxin was not lethal to the fly. However, when cholera toxin was co-administered with a pathogenic strain of *V. cholerae* carrying a chromosomal deletion of the genes encoding cholera toxin, death of the fly ensued. These findings suggest that additional virulence factors are required for intoxication of the fly that may not be essential for intoxication of mammals. Furthermore, we demonstrate for the first time the mechanism of action of cholera toxin in a whole organism and the utility of *D. melanogaster* as an accurate, inexpensive model for elucidation of host susceptibility to cholera.

## Synopsis

Cholera, the pandemic diarrheal disease caused by the gram-negative bacterium *Vibrio cholerae,* continues to be a major public health challenge in the developing world*.* Environmental studies have demonstrated a close association between *V. cholerae* and many species of arthropods, and insects have previously been implicated as vectors of this disease. Here researchers report the susceptibility of the fruit fly, *Drosophila melanogaster,* to oral *V. cholerae* infection through a process that exhibits many of the hallmarks of human disease. Furthermore, although ingestion of cholera toxin results in massive diarrhea in mammals, these researchers have found that ingestion of purified cholera toxin is not lethal to the fly. However, when co-ingested with a pathogenic strain of *V. cholerae* carrying a deletion of the cholera toxin genes, cholera toxin is lethal. These findings not only demonstrate the utility of *D. melanogaster* as an accurate, inexpensive model for elucidation of the host-pathogen interaction and identification of inhibitors of the action of cholera toxin; they also suggest that *V. cholerae* carries additional virulence factors that enable intoxication of an arthropod host. Based on these findings, the researchers suggest that the fly or a related arthropod may be a true host of *V. cholerae* in nature*.*

## Introduction

Cholera continues to be a major cause of morbidity and mortality in many parts of the world [1]. It is contracted through ingestion of contaminated food or water and is characterized by profuse diarrhea and vomiting. Cholera toxin, the primary determinant of this clinical syndrome, is an AB_5_-type exotoxin composed of an A subunit non-covalently bound to five B subunits, arranged in a rosette to form a lectin recognizing the GM_1_ ganglioside [2]. The mechanism by which cholera toxin enters intestinal epithelial cells and disrupts function has been studied extensively in cultured cells [3–7]. Prior to entry into the cell, the A subunit is proteolytically cleaved into a catalytic A_1_ subunit and an A_2_ subunit, whose role is to maintain the non-covalent association to the B subunit GM_1_ lectin. This lectin forms an association with GM_1_ gangliosides that are concentrated in lipid rafts within the cell membrane. Once bound to GM_1_, retrograde transport on lipid rafts delivers cholera toxin to the endoplasmic reticulum. The A_1_ subunit then dissociates from the toxin complex and exits the endoplasmic reticulum to ADP-ribosylate the stimulatory G protein subunit, G_sα_. The modified G_sα_ constitutively activates adenylyl cyclase, and levels of cAMP in intestinal epithelial cells rise. The consequent secretory diarrhea depends on opening of cAMP-responsive Cl^−^ channels and flow of Cl^−^ and water through the apical surface of the epithelial cell into the intestinal lumen. KCNN4, an intermediate conductance Ca^2+^-activated K^+^ channel of mammals, maintains K^+^ export through the basolateral aspect of the intestinal epithelial cell. Clotrimazole, which blocks the KCNN4 channel, has been shown to decrease cholera toxin-induced Cl^−^ secretion in both cultured mammalian cells and mice [8,9]. These results suggest that simultaneous basolateral export of K^+^ is required to maintain passage of Cl^−^ through basolateral K^+^/Cl^−^ cotransporters and apical Cl^−^ channels into the intestinal lumen.

The utility of *Drosophila melanogaster* as a model host for human pathogens is well-established [10–18]. In the natural environment, *Vibrio cholerae* is closely associated with arthropods [19–21], and many have suggested that insects serve as vectors [22–26] or reservoirs [27–29] of *V. cholerae*. Thus, we hypothesized that insects or related arthropods might serve as excellent model hosts of *V. cholerae.* To test this, we subjected the model insect *D. melanogaster* to oral *V. cholerae* infection. Here we demonstrate that *V. cholerae* infection of *D. melanogaster* exhibits the following parallels to human disease: (i) ingestion of *V. cholerae* produces an intestinally-localized, lethal infection in the fly that is dependent on cholera toxin; (ii) host susceptibility is dependent on Gsα, adenylyl cyclase, and the *Drosophila* KCNN4 channel homolog; and (iii) clotrimazole, an inhibitor of the human KCNN4 channel, protects the fly against infection. However, we have also found differences between *V. cholerae* infection of mammals and flies. Ingestion of cholera toxin alone is sufficient to cause severe secretory diarrhea in humans and model mammals [30–33]. In contrast, in the fly, we have found that ingestion of cholera toxin is lethal only when pathogenic isolates of *V. cholerae* are ingested in tandem. Our findings not only demonstrate the utility of the fly as a model host for *V. cholerae* infection, but also suggest that the *V. cholerae* genome contains virulence factors specifically required for infection of non-mammalian hosts such as the fly.

## Results/Discussion

### Ingestion of *V. cholerae* Results in Lethal Infection of *D. melanogaster*

**Figure 1:**
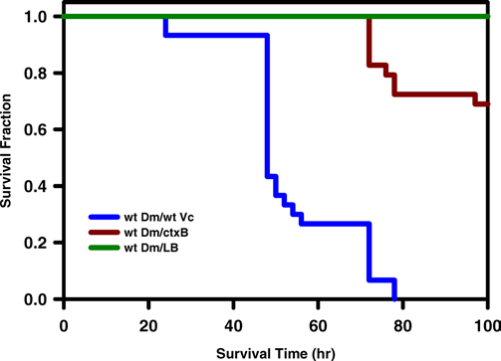
The Genes Encoding Cholera Toxin Are Required for Lethal *V. cholerae* Infection of *Drosophila* Fractional survival of wild-type Oregon R flies (wtDm) fed LB alone (LB), wild-type *V. cholerae* (wtVc), or a *V. cholerae* Δ*ctxB* mutant (ctxB). Ten adult flies (five males and five females), 3–5 d following eclosion were used. Log-rank test analysis demonstrated a statistically significance difference in survival of wild-type *V. cholerae* infected flies and *V. cholerae* Δ*ctxB* mutant infected flies (*p* < 0.0001).

To test the utility of *D. melanogaster* as a model host for *V. cholerae,* flies were fed either Luria-Bertani (LB) broth alone or inoculated with *V. cholerae*. Consumption of this growth medium by the fly was documented on multiple occasions by addition of blue dye. Using this experimental design, wild-type flies fed LB broth alone survived for 5 d and could be maintained for up to 2 wk if a larger volume of LB broth was provided. In contrast, flies fed LB inoculated with *V. cholerae* expired after 3 d regardless of the amount of volume provided (Figure 1). Similar observations were made for the Canton-S wild-type strain of *D. melanogaster* and for several *D. melanogaster* strains carrying benign marker mutations (unpublished data).

### *V. cholerae* Is Able to Multiply within the Fly

**Figure 2:**
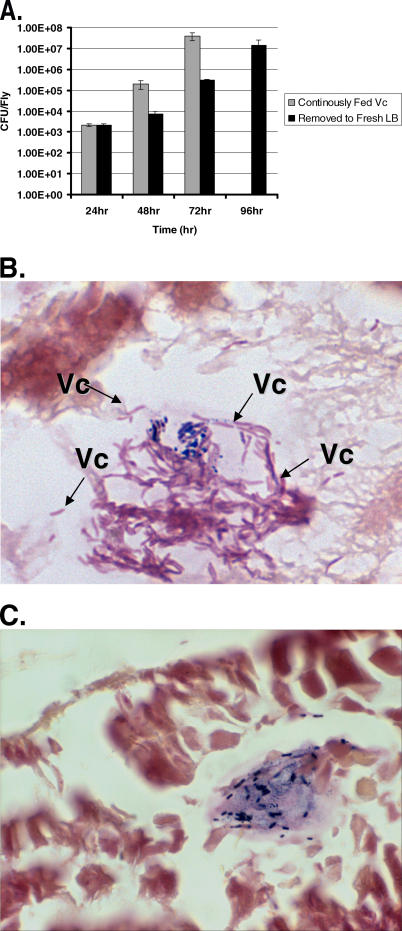
*V. cholerae* Multiplies within the Gut of the Fly following Infection (A) Colony counts were assayed at 24-h time points from flies infected with *V. cholerae.* Grey bars indicate CFU per fly obtained from flies fed *V. cholerae* continuously, while black bars depict CFU per fly for flies fed *V. cholerae* for 24 h and then removed to a sterile, fresh LB solution. (B) Section of the midgut of a fly harvested 48 h after introduction to medium containing *V. cholerae*. Arrows labeled with Vc point to clusters of slender, curved gram negative *V. cholerae* (pink) present in the lumen of the midgut of the infected fly. Occasional gram positive bacteria (violet), which represent the endogenous flora of the gut, are also present. (C) Section of the midgut of a fly harvested 48 h after introduction to LB alone. Only endogenous gram positive bacteria (violet) could be observed in the intestines of flies fed sterile LB broth.

Once ingested by a model mammalian host, *V. cholerae* is able to multiply within the intestinal compartment [34]. In the experimental model presented above, flies were continuously fed *V. cholerae.* While this type of infection is rapidly lethal, it does not distinguish between bacterial accumulation and bacterial colonization and multiplication. To test whether *V. cholerae* was able to persist and multiply within the fly, we measured *V. cholerae* colony-forming unit (CFU)/fly over time in flies continuously fed LB inoculated with *V.* cholerae and in flies first fed LB inoculated with *V. cholerae* for 24 h and then transferred to a vial containing sterile LB broth. At 24 h, flies in both groups harbored equivalent numbers of *V. cholerae.* As shown in Figure 2A, flies exposed continuously to LB inoculated with *V. cholerae* expired after 3 d when the burden of *V. cholerae* reached 3.93 × 10^7^ CFU/fly. Over the course of 4 d, numbers of *V. cholerae* also increased in flies removed from contaminated food, albeit at a slower rate than flies continuously exposed to *V. cholerae.* The number of *V. cholerae* required to bring about death was similar in both groups. These results suggest that *V. cholerae* is able to colonize and multiply within the fly in the absence of continued ingestion.

### *V. cholerae* Remains Localized to the Fly Gut following Ingestion

During human infection, *V. cholerae* remains localized to the intestine, causing systemic disease through the action of cholera toxin. To determine whether *V. cholerae* also remained localized to the *Drosophila* gut, whole flies fed either sterile LB or the *V. cholerae*/LB mixture were processed into 5-μm thick histologic sections, stained, and examined. Many slender, comma-shaped, gram-negative rods were found within the midgut of *V. cholerae*-infected flies (Figure 2B). Although concentrated in the midgut, *V. cholerae* were also found in other regions of the gut. Careful histologic analysis of all tissues revealed no *V. cholerae* outside the fly alimentary tract. Interestingly, the intestines of flies fed both sterile LB, and LB inoculated with *V. cholerae* contained gram-positive rods (Figure 2C). These most likely represent the commensal flora of our laboratory flies.

### Cholera Toxin Is a Virulence Factor in *V. cholerae* Infection of the Fly

We hypothesized that, as is the case in human disease, cholera toxin secreted from *V. cholerae* within the fly gut was responsible for death. To test this hypothesis, a *V. cholerae* mutant harboring a deletion in the *ctxB* gene was constructed and fed to wild-type flies [35]. The Δ*ctxB* mutant was significantly less virulent in the fly model of cholera, demonstrating that cholera toxin is the primary virulence factor in *V. cholerae* infection of both flies and humans (Figure 1). Although flies fed a Δ*ctxB* mutant survived several days longer than flies fed wild-type *V. cholerae,* they still died prematurely. Thus, we hypothesize that, in the absence of cholera toxin, other virulence factors contribute to death of the fly.

### *V. cholerae*-Infected Flies Lose Weight Prior to Death

**Figure 3:**
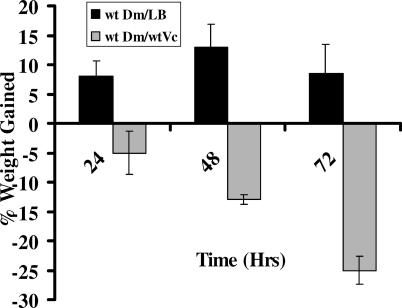
Ingestion of *V. cholerae* Induces *Drosophila* Weight Loss Fraction of initial weight gained by wild-type flies (wt Dm) fed either LB alone (LB) or *V. cholerae* (wt Vc). Error bars represent the standard deviation based on three measurements.

Cholera victims may lose 10% or more of their body weight due to dehydration as a result of secretory diarrhea [36]. If cholera toxin acts *via* a similar mechanism in the fly, weight loss should also occur during infection of the fly. To test this, flies fed either LB alone or LB inoculated with *V. cholerae* were weighed on a daily basis. Over the course of 3 d, flies fed *V. cholerae* lost approximately 25 % of their initial body weight, while flies fed LB alone showed a small weight gain (Figure 3). These results support the hypothesis that flies, like humans, become dehydrated during *V. cholerae* infection. However, we cannot exclude other causes of weight loss such as a decreased food intake or altered metabolic activity.

### G-sα60A, Adenylyl Cyclase, and SK Channel Mutants Are Resistant to Lethal *V. cholerae* Infection

**Figure 4:**
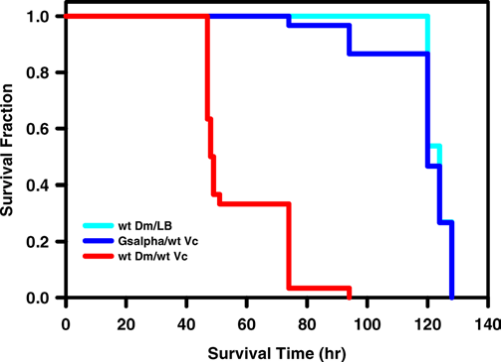
A *G-sα60A^R60^* Mutant Strain Is Resistant to Lethal *V. cholerae* Infection Fractional survival over time of wild-type flies (Oregon R; wt Dm) and *G-sα60A^R60^* mutant flies [44] that were fed either LB alone or LB inoculated with wild-type *V. cholerae* (wt Vc). In these experiments and those illustrated in Figures 5 and 6, ten 3- to 5-d-old adult flies (five males and five females) were infected, and all experiments were performed in triplicate. Log-rank test analysis demonstrated a statistically significant difference in survival of wild-type flies fed wild-type *V. cholerae* and *G-sα60A^R60^* mutant flies fed wild-type *V. cholerae* (*p* < 0.0001).

**Figure 5:**
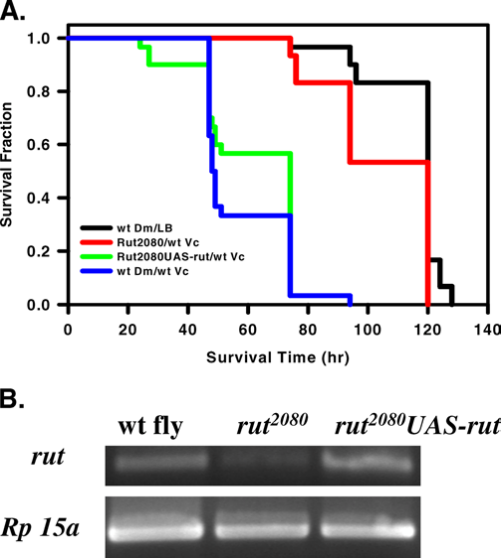
A *rut^2080^* Mutant Strain Is Resistant to Lethal *V. cholerae* Infection (A) Fractional survival over time of wild type flies, *rut^2080^* mutant flies [47], and *rut^2080^* *UAS-rut^+^* fed LB inoculated with *V. cholerae* (wt Vc). Wild-type flies fed LB broth alone were included as a control. Log-rank test analysis demonstrated a statistically significant difference in the survival of wild-type flies fed wild-type *V. cholerae* and *rut^2080^* mutant flies fed wild-type *V. cholerae* (*p* < 0.0001). (B) RT-PCR amplification of *rutabaga* transcripts in wild-type (WT), *rut^2080^*, and *rut^2080^ UAS-rut* ^+^ flies. The ribosomal protein *rp15a* was used as a constitutively transcribed control.

**Figure 6:**
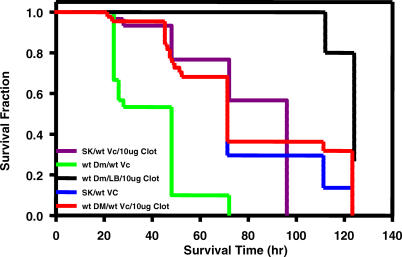
SK Mutant *Drosophila a*nd Clotrimazole-Treated Wild-Type Flies Display Partial Resistance to Lethal *V. cholerae* Infection Fractional survival over time of wild-type (wt Dm) or *SK* mutant ((WH}SK^f07979^) flies fed either wild-type *V. cholerae* alone or combined with 10 μg/ml clotrimazole (10 μg Clot). Log-rank test analysis demonstrated a statistically significant difference in survival of wild-type flies fed wild-type *V. cholerae* and *SK* mutant ((WH}SK^f07979^) flies fed wild-type *V. cholerae* (*p* < 0.0001). There was also a statistically significant difference in survival of wild-type flies fed wild-type *V. cholerae* and wild-type flies fed wild-type *V. cholerae* combined with 10 μg/ml clotrimazole (*p* < 0.0001).

Cell culture-based studies have shown that G_sα_, adenylyl cyclase, and the KCNN4 channel play an important role in *V. cholerae*-induced Cl^−^ secretion by intestinal epithelial cells [9,37,38]. We asked whether these same factors might be required for susceptibility of *Drosophila* to *V. cholerae* infection by examining the susceptibility of *Drosophila* strains bearing mutations in the genes encoding G-sα60A, the adenylyl cyclase rutabaga, or the SK channel, a Ca^2+^-sensitive K^+^ channel that is the closest *Drosophila* homolog of the human KCNN4 channel. As shown in Figures 4 and 5A, mutation of *G-sα60A* and *rutabaga* provided nearly complete protection against *V. cholerae* infection. Mutation of *Sk* provided only partial protection. This may be the result of persistent, albeit reduced expression of the SK channel in this mutant or of additional mechanisms that facilitate Cl^−^ secretion in the fly (Figure 6). Importantly, we confirmed that the additional independently generated mutant alleles for G-sα60A, rut, or SK listed in Table 1 had similar effects on *V. cholerae* susceptibility, indicating that mutations in these genes, rather than other differences in genetic background, caused the observed phenotypes.

**Table 1:**
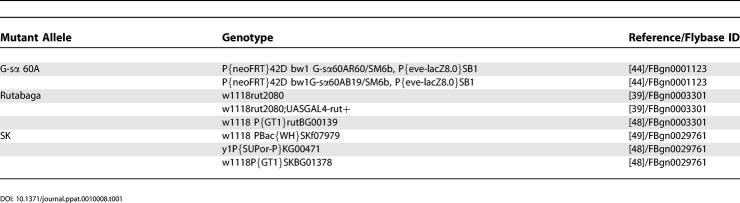
*Drosophila* Alleles Used in Mutant Studies

DOI: 10.1371/journal.ppat.0010008.t001

In preparation for genetic rescue of the *rut* mutant phenotype using the GAL4/UAS binary expression system, a *rut^2080^* strain homozygous for a *UAS-rut* ^+^ transgene insertion on the second chromosome was obtained and assayed for susceptibility to *V. cholerae* infection [39]. Unexpectedly, these flies were susceptible (Figure 5A)*.* To ascertain the basis of this susceptibility, we assayed levels of *rut* transcript in wild-type, *rut^2080^*, and *rut^2080^;UAS-rut* ^+^ flies by RT-PCR. As shown in Figure 5B, *rut* transcription was greatly reduced in the *rut^2080^* mutant, but the *rut^2080^;UAS-rut* ^+^ flies had transcript levels comparable to those of wild-type flies. PCR analysis confirmed the presence of the *rut^2080^* insertion in both strains. Thus, we conclude that the *UAS-rut* transgene is transcribed in the absence of Gal4, presumably by regulation from an adjacent genomic element. Furthermore, we conclude that susceptibility of *rut* mutant flies to *V. cholerae* infection is rescued by restoration of wild-type levels of the *rutabaga* transcript.

### Clotrimazole Protects *V. cholerae*-Infected Flies against Death

Because clotrimazole abrogates the *V. cholerae*-induced secretory diarrhea in mammals by inhibiting K^+^ transport through KCNN4 channels, we postulated that co-administration of clotrimazole with *V. cholerae* might also block K^+^ transport through the *Drosophila* SK channel and, therefore, protect wild-type flies against death. Figure 6 shows that this was indeed the case. However, co-administration of clotrimazole had no effect on survival of *SK* mutant flies, suggesting that clotrimazole is, in fact, exerting its effect by interaction with the SK channel (Figure 6).

### A Factor Carried by Pathogenic *V. cholerae* Is Required for Intoxication of the Fly by Cholera Toxin

**Figure 7:**
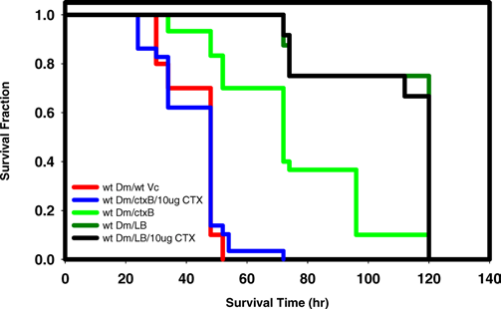
A Bacterial Factor Is Required for Intoxication of the Fly by Cholera Toxin Fractional survival over time of wild-type flies fed LB alone, wild-type *V. cholerae,* or a *V. cholerae*Δ*ctxB* mutant (ctxB) either with or without 10 μg/ml purified cholera toxin. Log-rank test analysis demonstrated a statistically significant difference in the survival of wild-type flies fed a *V. cholerae* Δ*ctxB* mutant (ctxB) alone and those fed a *V. cholerae* Δ*ctxB* mutant (ctxB) combined with purified cholera toxin (*p* < 0.0001).

Ingestion of cholera toxin is sufficient to cause massive intestinal fluid accumulation and diarrhea in mammals [30–33]. Thus, we predicted that ingestion of purified, active cholera toxin alone would result in death of the fly. Remarkably, ingestion of LB containing as much as 100 μg/ml of cholera toxin did not alter survival of the fly (unpublished data). We questioned whether the presence of *V. cholerae* itself might be required for intoxication of the fly by cholera toxin. To test this, we fed LB containing both cholera toxin and a *V. cholerae* Δ*ctxB* mutant to flies. As shown in Figure 7, ingestion of cholera toxin in the presence of the Δ*ctxB* mutant *V. cholerae* resulted in death of the flies at rates similar to those of flies infected with wild-type *V. cholerae* alone. This suggested to us that an unknown bacterial factor might be required for intoxication of the fly by cholera toxin. To determine whether this factor might be specific to pathogenic isolates of *V. cholerae,* we fed LB containing cholera toxin and one of several non-toxigenic environmental isolates of *V. cholerae* to flies. In each case, there was no significant difference in survival between flies fed *V. cholerae* alone and those fed *V. cholerae* combined with cholera toxin. To test whether this cholera toxin-potentiating factor was carried on the CTXΦ, we combined cholera toxin with Bengal2, a pathogenic strain of *V. cholerae* carrying a deletion of the CTXΦ. This mutant was also able to provide the fly-specific virulence factor (unpublished data). Thus, this factor is not carried on the CTXΦ. These experiments suggest that pathogenic *V. cholerae* possess a virulence factor or factors that are essential for intoxication of arthropods but not mammals by cholera toxin.

### Implications of this Model for the Study, Treatment, and Ecology of Cholera

We have demonstrated surprising parallels in the mechanism of *V. cholerae*-mediated death of man and the model arthropod *D. melanogaster*. Cholera toxin is the primary virulence factor in both infections. While the mechanism of cholera toxin has previously been elucidated in cultured intestinal epithelial cells, we present the first evidence that this mechanism is also operative in whole organisms. Furthermore, this model system will have wide-ranging applications to the study of this devastating disease. Due to the expense and labor involved in mammalian genetic screens, little is known about the host factors that govern susceptibility to cholera. Because lethal oral infection of the fly requires no manipulation by the experimentalist and has an easily measured outcome, the fly provides a powerful tool to be used in large-scale genetic screens for host susceptibility factors and bacterial virulence factors. The current mainstay of cholera therapy consists of administration of oral or intravenous water and ions until the infection is overcome by antibiotics and /or the innate immune system. An inhibitor of the secretory diarrhea caused by cholera toxin would be a potentially life-saving adjuvant to this therapy. We have shown here that oral agents can block the action of cholera toxin in the fly. Thus, this model will also facilitate screens of combinatorial chemical libraries for inhibitors of cholera toxin and secretory diarrhea. Finally, these studies highlight a host-pathogen interaction that could easily occur in nature. Close contact between *V. cholerae* and arthropods has been documented and is likely more frequent than that between *V. cholerae* and humans [19,40–42]. In fact, environmental studies have demonstrated that common house flies carry *V. cholerae* in endemic areas [22–25]. In this work, we have presented evidence that pathogenic *V. cholerae* carry virulence factors that are essential for intoxication of the fly but not mammals. Thus, we present the provocative hypothesis that the pathogenic program of *V. cholerae* may have evolved for an arthropod rather than for us.

## Materials and Methods

### Bacterial strains, fly strains, and growth media.

MO10, a *V. cholerae* O139 clinical isolate, and mutants derived from this strain were used in all experiments [43]. All fly strains were reared at room temperature on standard *Drosophila* media. The wild-type OregonR fly strain was used for most studies. Gsα, *rut,* and *Sk* experiments utilized mutant fly lines harboring *G-sα 60A^R60^*, a loss-of-function allele that reduces the cAMP concentration 4- to 5-fold in larvae [44], *rut^2080^*, an enhancer trap element in the 5^′^ flanking region of the *rut* gene [45], and PBac(WH}SK^f07979^ , respectively (Table 1). The *rut^2080^* and *rut^2080^;UAS-rut^+^* fly lines were generously provided by Ron Davis. The presence of the *rut^2080^* mutant allele was confirmed by PCR amplification of a portion of the insertion element for both lines. Additionally, fly lines carrying G-sα60A^B19^, P(EP}rut^EP399^ or P(GT1}rut^BG00139^, and P(SUPor-P}KG00471 or P(GT1}SK^BG01378^ were used to confirm the results of experiments with the *G-sα60A^R60^* , *rut^2080^*, and PBac(WH}SK^f07979^ mutant fly strains, respectively (Table 1). Fly lines other than those noted were obtained from the Bloomington *Drosophila* Stock Center (Bloomington, Indiana).

### *V. cholerae* mutant construction.

The *V. cholerae* Δ*ctxB* mutant, harboring a 321 bp deletion in the *ctxB* gene (VC1456) was constructed by double homologous recombination according to previously described protocols [35]. The deletion removed all but 11 amino acids remaining at the amino-terminus of the protein and the terminal stop codon.

### Survival of *Drosophila* following *V. cholerae* infection.

Ten wild-type Oregon-R adult flies were placed in each of three vials containing a cotton plug saturated with Luria-Bertani (LB) broth either alone or inoculated with 10^8^ CFU/ml of *V. cholerae* O139 strain MO10 or another strain as noted in the text [46]. Viable flies were counted at 24-h intervals. Reproducibility of all survival curves was confirmed in at least three independent experiments, and log-rank tests were used to determine statistical significance.

### Histological studies.

Flies were fed either LB inoculated with *V. cholerae* or LB alone for 48 h, and then anesthetized and fixed in formalin for 48 h prior to processing. Flies were processed on a tissue processor (Leica ASP 300, Wetzlar, Germany) and embedded in paraffin. The embedded flies were sectioned into 5-μm ribbons, which were placed on positively charged glass slides, baked at 65 °C overnight, and gram stained.

### Weight loss measurements.

Sets of ten female flies were weighed and then transferred to fly vials containing either LB alone or LB inoculated with *V. cholerae.* Flies, housed in thin-walled Eppendorf tubes, were weighed 24 and 48 h after transfer, using a precision balance (Mettler Toledo AG204, Columbus, Ohio). All experiments were performed in triplicate, and the average ratios of final to initial weight were calculated.

### Quantification of *V. cholerae* within flies.

To determine whether *V. cholerae* was able to colonize and multiply within the fly, flies fed either LB alone or LB inoculated with *V. cholerae* were anesthetized, removed from vials, and homogenized in LB broth at 24-h intervals. Particulates were pelleted, and dilutions of the resulting supernatants were plated on LB-agar supplemented with streptomycin (100 mg/ml). In all cases, no colonies were obtained from LB-fed flies.

### RT-PCR.

Total RNA was extracted from five flies using the Trizol reagent (Gibco BRL, San Diego, California, United States). Prior to RT-PCR amplification, total RNA was DNAase I-treated (Ambion, Austin, Texas, United States) for 30 min at 37 °C. DNAse I was inactivated using the DNAse inactivation reagent (Ambion). RT-PCR was performed in two steps using Superscript II RT (Gibco BRL) to obtain cDNA and Taq to perform PCR. The following primer pairs were used: *rut* (5′-GATCCAGGATGAGAACGA-3′, 5′-CGGAGACACAATAGTAACAGTC-3′) and *Drosophila* ribosomal protein 15a (5′-CGTTTGCGTGACGGTCGTGT-3′, 5′-GCCGAGAATTTTGCCTCCCAA-3′).

### Fly intoxication with purified cholera toxin.

Adult Oregon-R flies 3–5 d old were fed cholera toxin diluted to the specified concentrations in LB broth. Overnight cultures containing *V. cholerae* strains were also added to the mixture in a 1:10 dilution where specified. Flies were monitored at 24-h time intervals until death. Survival of flies was plotted against time using Kaplan-Meier plots, and a log-rank test was performed to determine statistical significance.

## Abbreviations

CFU: colony-forming units
LB: Luria-Bertani broth
